# Oral ulcers in hematological malignancy patients undergoing chemotherapy: is it chemotherapy or neutropenia?: a case report and review of the literature

**DOI:** 10.1186/s13256-025-05180-8

**Published:** 2025-03-17

**Authors:** Fatima AlZahraa Al Beesh, Nafiza Martini, Siham Suleiman, Abeer Aljoujou

**Affiliations:** 1 Department of Oral Medicine, Faculty of Dentistry, University of Damascus, Damascus, Syrian Arab Republic; 2 Faculty of Medicine, University of Damascus, Damascus, Syrian Arab Republic; 3 Stemosis for Scientific Research, Damascus, Syrian Arab Republic; 4 Department of Hematology-Oncology, Faculty of Medicine, University of Damascus, Damascus, Syrian Arab Republic

**Keywords:** Neutropenic ulcer, Neutropenia, Oral ulcer, Chemotherapy-induced neutropenia

## Abstract

**Background:**

Chemotherapy can cause oral complications either directly, by inducing mucosal degeneration, or indirectly, through myelosuppression leading to neutropenia. Neutropenia, a common side effect, is often associated with multiple oral ulcers.

**Case presentation:**

A 39-year-old Arabic Syrian man with acute myelogenous leukemia developed neutropenia following his initial chemotherapy course, resulting in oral ulcers. A complete blood count confirmed chemotherapy-induced neutropenia, and the clinical presentation supported the diagnosis of neutropenic ulcers. The patient’s chemotherapy regimen was temporarily halted, and a topical corticosteroid paste containing triamcinolone acetonide was applied three times daily for 7 days. This treatment led to significant regression of the ulcers. The patient provided written informed consent after receiving a detailed explanation of the study’s objectives, procedures, and privacy considerations.

**Conclusion:**

Topical corticosteroid treatment effectively promoted significant regression of neutropenic ulcers in this case.

## Introduction

Hematological malignancies comprise a diverse group of cancers arising from the uncontrolled proliferation and malignant transformation of hematopoietic cells. These malignancies vary significantly in incidence, etiology, prognosis, and mortality rates. Globally, they rank as the fifth most prevalent cancer type, accounting for approximately 6.5% of all cancer cases and the second leading cause of cancer-related mortality [1–7]. They are broadly classified into myelocytic and lymphocytic subtypes on the basis of cell lineage [3, 5], with leukemias and lymphomas being the most common types worldwide [1, 2, 6, 7].

The management of hematological malignancies depends on factors such as disease subtype, patient age, and comorbidities. Treatment options include chemotherapy, radiotherapy, stem cell transplantation, and supportive care [8–10]. Among these, chemotherapy remains the primary treatment modality, exerting cytotoxic effects on rapidly dividing cells by disrupting DNA and RNA synthesis, causing DNA damage, and interfering with mitotic spindle formation [11–17]. It employs various agents, including alkylating agents, antimetabolites, antimitotics, and certain antibiotics [9, 11]. However, owing to its nonselective nature, chemotherapy not only targets malignant cells but also affects rapidly proliferating normal cells, particularly within the hematopoietic system, leading to a range of adverse effects [11–14, 16–20].

One of the most serious complications of chemotherapy is neutropenia, resulting from bone marrow suppression. Neutropenia increases the susceptibility to infections and other complications [21–26], as neutrophils play a crucial role in immune defense, acute inflammatory responses, and tissue repair [27–31]. In patients with hematological malignancies receiving myelosuppressive chemotherapy, the oral mucosa is especially vulnerable to toxicity owing to its high cellular turnover, distinct microbiome, presence of odontogenic infection foci, and exposure to physiological trauma [13, 18, 32–36]. The combination of neutropenia and immune suppression disrupts oral hemostasis, predisposing patients to oral complications such as mucositis and ulceration [13, 32–34], which significantly impact quality of life and treatment adherence [35]. This article explores the etiology and management of neutropenic ulcers, presenting a case report of a patient with acute myelogenous leukemia who developed multiple painless oral ulcers following a chemotherapy-induced neutropenia. Future research is needed to develop more effective strategies for preventing and managing oral mucositis, ultimately improving patient outcomes and chemotherapy efficacy.

## Case presentation

A 39-year-old Syrian male of Arabic descent, diagnosed with acute myelogenous leukemia (AML), underwent his initial chemotherapy at Al-Bairouni University Hospital, Damascus’s primary oncology center. The induction regimen followed the standard 7 + 3 protocol, consisting of cytarabine for 7 days and anthracycline for 3 days. Three days after initiating chemotherapy, the patient developed moderate neutropenia [absolute neutrophil count (ANC): 0.9 × 10^9^/L]. Complete blood count (CBC) also revealed a reduced red blood cell count (2.31 × 10^12^/L) and platelet count (11 × 10^9^/L).

**Fig. 1:**
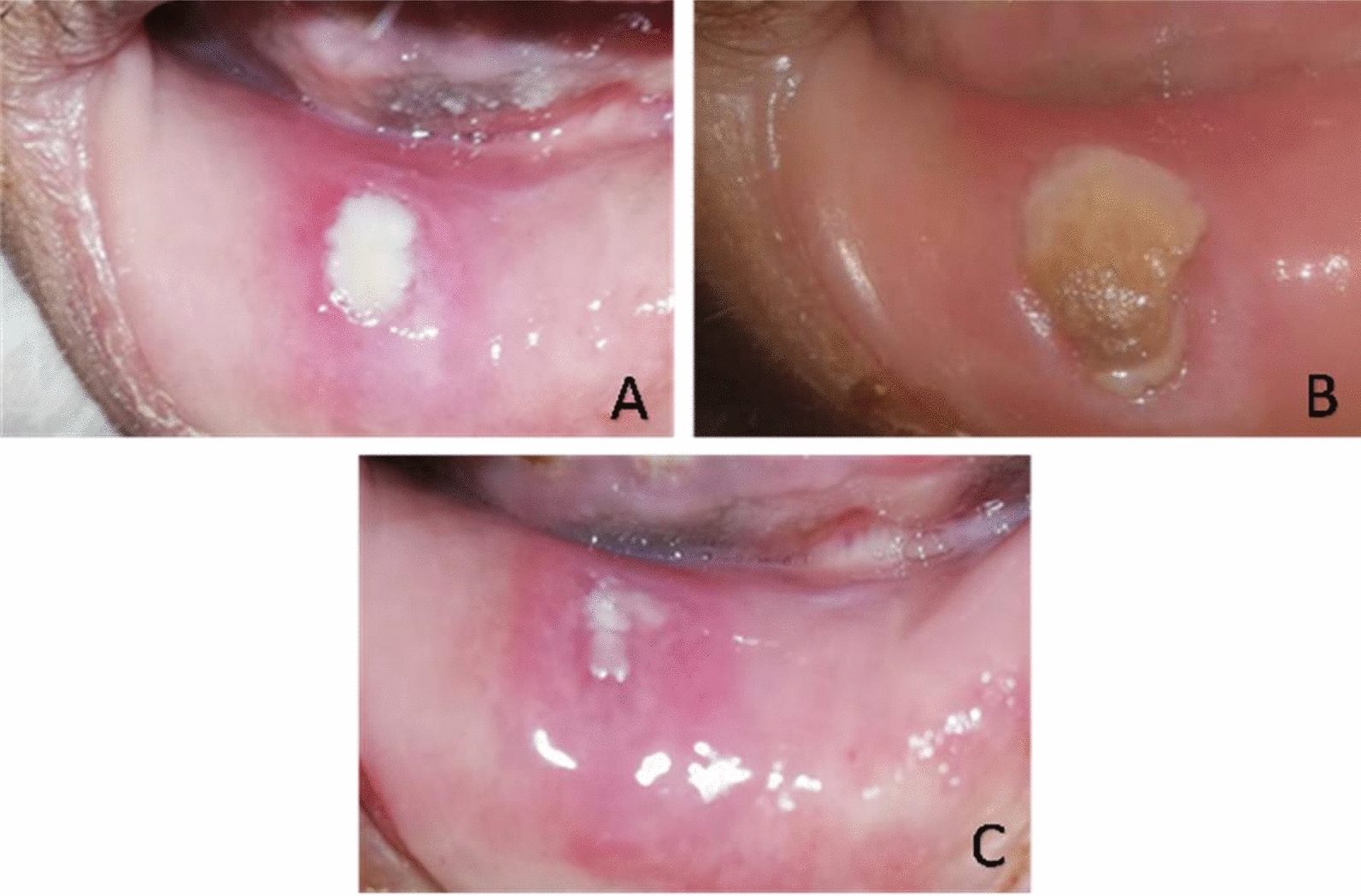
Displays an ulcer located on the lower lip. **A** The ulcer after 5 days of neutropenia with a pseudo-fibrinous membrane and an erythematous halo. The ulcer appears nonhemorrhagic, painless, and measures approximately 1.5 cm × 1 cm in size. **B** The ulcer on the lower lip after 12 days of neutropenia. There is evident growth in the size of the ulcer and the presence of necrotic tissue. **C** Reduction in the size of the ulcer on the lower lip, taken 7 days after initiating the topical treatment

**Fig. 2:**
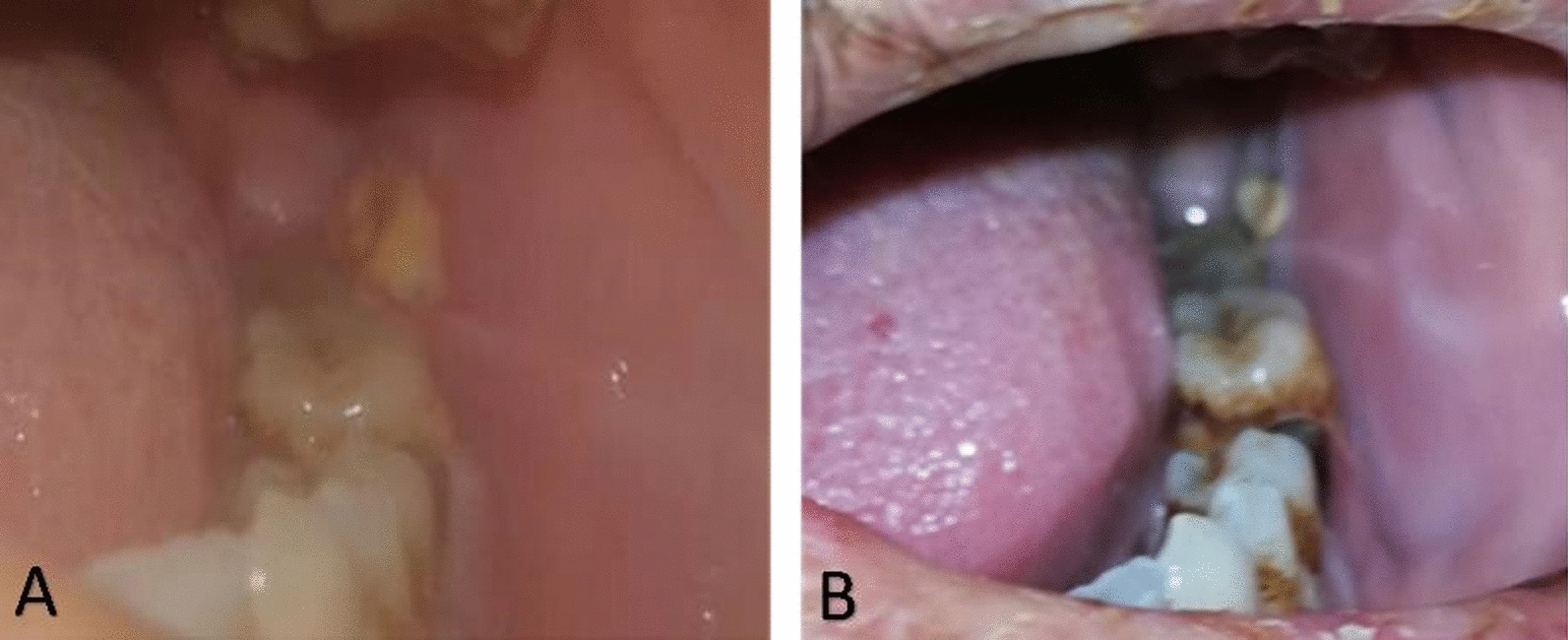
**A** A new necrotic ulcer which showed after 12 days of neutropenia, and is located on the left buccal mucosa. The ulcer is painless, non-bleeding, and measures approximately 1 cm × 1 cm in size. **B** A notable decrease in the size of the ulcer on the left buccal mucosa

Two days after neutropenia onset, a clinical oral examination identified a painless ulcer covered by a pseudo-fibrinous membrane on the lower labial mucosa. Initially measuring 1.5 cm × 1 cm, the ulcer exhibited an erythematous halo and edema (Fig. 1A). After 1 week, it expanded to 2 cm × 1.5 cm, developing necrotic edges while remaining painless (Fig. 1B). In addition, a 1 cm × 1.5 cm necrotic ulcer was observed on the left buccal mucosa (Fig. 2A).

**Fig. 3:**
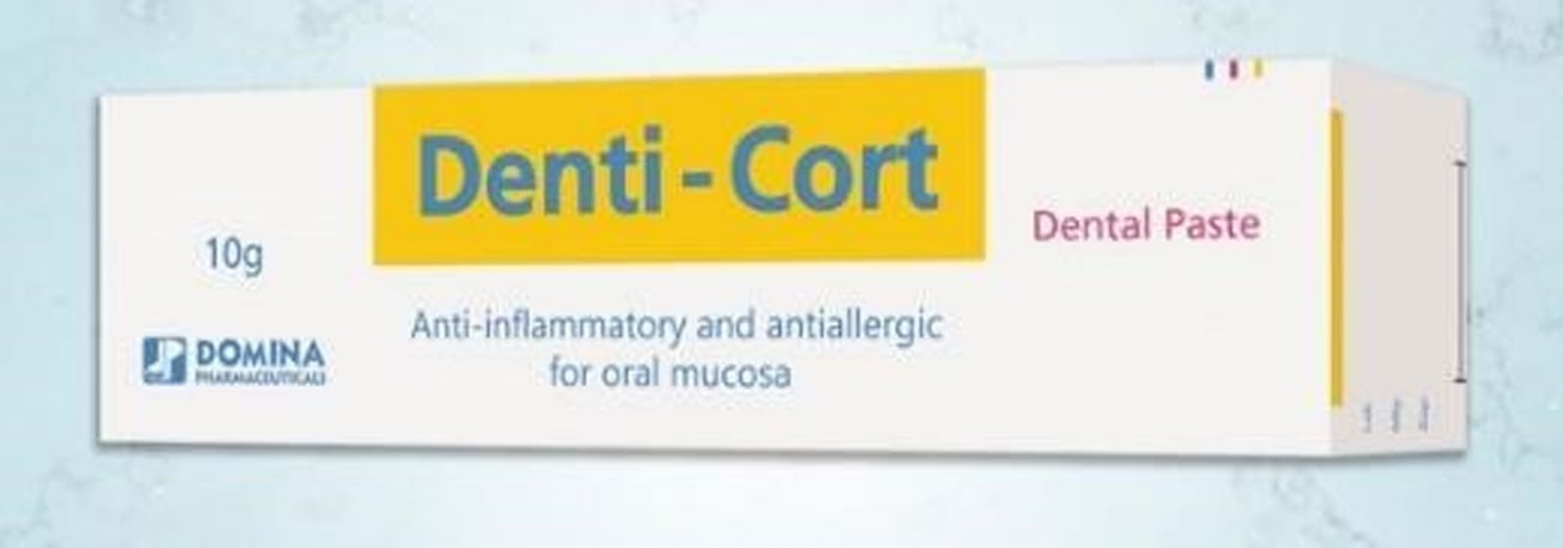
Shows the topical medication containing triamcinolone acetonide in Orabase, which was utilized in the treatment

To manage the ulcers, the patient was prescribed topical corticosteroids and antiseptic mouth rinses [37]. Chlorhexidine 0.02% was used as a mouthwash twice daily, and a topical paste containing triamcinolone acetonide (Denti-Cort) in Orabase was applied three times daily for 7 days (Fig. 3). Upon reevaluation, the ulcers showed significant regression, with no pain or bleeding (Figs. 1C and 2B).

The patient provided written informed consent after receiving a thorough explanation of the study objectives, procedures, and privacy considerations.

## Discussion

The introduction of chemotherapy in the 1940s led to the recognition of mucositis as a significant adverse effect. Initially termed stomatitis, its pathogenesis was not fully understood until six decades later, prompting the adoption of the term “mucositis” in 2007 to describe lesions caused by cytotoxic chemotherapy [38]. Mucositis can affect multiple segments of the gastrointestinal tract, including the oral cavity, where it presents as oral mucositis (OM). OM typically develops 4–16 days after initiating a high-dose chemotherapy and persists for 10–14 days post-treatment [13, 35]. It is characterized by inflammation, epithelial damage, ulceration, and impaired cell regeneration, leading to mucosal atrophy. Clinically, OM manifests as erythema, a burning sensation, swelling, and mucosal desquamation, ultimately leading to ulcer formation [12, 13, 19, 35, 38, 39]. These crater-like ulcers, covered by pseudo-fibrinous tissue with indistinct edges, indicate complete epithelial loss and partial soft tissue involvement [13, 40]. OM is a common chemotherapy complication, affecting approximately 40% of patients, often resulting in severe pain, functional impairment, and treatment delays, which negatively impact quality of life [35, 38].

The development of mucositis occurs through a five-phase process, influenced by cytokine activity, chemotherapy-induced cytotoxicity, oral microbiome composition, and bone marrow status [38, 41, 42].

1. Inflammatory/vascular (initiation) phase: Chemotherapy-induced damage to epithelial and submucosal cells triggers the release of reactive oxygen species (ROS). ROS cause DNA damage, apoptosis, and vascular impairment leading to early mucosal injury [35, 38].
2. Epithelial phase: Free radicals disrupt DNA strands, inducing apoptosis. Activation of nuclear transcription factor kappa B (NF-kB) stimulates the release of pro-inflammatory cytokines, further impairing cell regeneration and mucosal integrity [35, 38].
3. Signal formation and transmission: Transcription factors activate the ceramide apoptosis pathway, exacerbating programmed cell death. Macrophages and matrix metalloproteinases further degrade tissue structure, while tumor necrosis factor-alpha (TNFα) accelerates mucosal damage [35].
4. Ulcerative phase: Damage to oral epithelial stem cell results in ulcer formation, particularly during periods of neutropenia. These ulcers are highly susceptible to secondary infection, increasing the risk of bacteremia or sepsis [35, 38].
5. Healing phase: Epithelial regeneration and microbiome restoration occur around day 15 or 16 post-chemotherapy, coinciding with peripheral blood recovery [35].

In chemotherapy patients, oral ulcers can result from neutropenia, direct chemotherapy toxicity, or infections [37]. Neutropenic ulcers are particularly concerning, as studies indicate a high prevalence of oral ulcers in neutropenic patients undergoing chemotherapy [43]. These ulcers are deep, irregularly shaped, and covered by a yellow pseudo-fibrin membrane, often exhibiting necrosis. While pain is a common symptom, some patients may experience reduced pain sensitivity due to chemotherapy-induced peripheral neuropathy [37, 40, 42, 44–46].

The diagnosis of OM is primarily clinical, based on characteristic ulcerative lesions, erythema and mucosal atrophy. In cases where superinfection is suspected, complementary diagnostic tests—such as cultures for candida, polymerase chain reaction (PCR) for herpes simplex virus, and bacterial cultures—may be necessary to confirm secondary infections. In addition, a CBC can be useful in identifying neutropenia and thrombocytopenia, both of which may predispose patients to more severe OM manifestations [42]. In this patient’s case, the diagnosis of neutropenic ulcers was established on the basis of the clinical presentations and laboratory findings, confirming neutropenia.

Currently, there is no standardized protocol for the management of OM; however, treatment generally consists of local and systemic interventions aimed at pain relief, inflammation control, infection prevention, and tissue regeneration [13, 35, 42, 47, 48]

1. Pain management
  Topical or systemic analgesics: lidocaine gel is commonly used to provide localized pain relief in ulcerated regions [47].
  Cryotherapy: the application of ice chips during chemotherapy administration may reduce pain and inflammation by decreasing mucosal blood flow and limiting chemotherapy exposure to the oral epithelium [42].
2. Anti-inflammatory and healing agents
  Topical corticosteroids (e.g., triamcinolone acetonide) help reduce inflammation and promote healing [47].
  Biological response factors such as granulocyte colony stimulating factor (G-CSF) have been investigated for their potential role in mucosal regeneration [42].
  Antioxidants (e.g., glutamine and honey-based formulations) may facilitate mucosal repair by reducing reactive oxygen species [42].
3. Antimicrobial and infection control strategies
  Antiseptic mouthwashes (e.g., chlorhexidine 0.02%) help prevent secondary infections, as chlorhexidine exhibits bactericidal, fungicidal, and virucidal properties [48].
  Topical or systemic antibiotics may be required if bacterial superinfection is suspected.
  Antifungal or antiviral medications are used when fungal or viral infections are present.
4. Non-pharmacological interventions
  Low level laser therapy (LLLT) has shown promise in reducing severity of OM and promoting healing [42].
  Oral care protocols (soft-bristled toothbrushes, saline rinses, and adequate hydration) are essential in preventing OM progression [42].
5. Management of neutropenic ulcers
  Corticosteroids (topical or systemic) help reduce inflammation and promote healing.
  Antiseptics and antibiotics are often necessary to prevent infections, particularly in immunocompromised patients.
  Neutrophil recovery is a critical factor, as ulcers typically heal once neutrophil counts normalize [49, 50].

In this case, the patient’s oral ulcer characteristics were consistent with neutropenic ulcers, and treatment with topical corticosteroid led to a significant improvement. The absence of pain was likely attributable to chemotherapy-induced neuropathy [13]. The prescribed regimen included chlorhexidine 0.02% mouthwash (twice daily) for antisepsis and infection prevention, and triamcinolone acetonide-based topical paste (Denti-Cort) (three times daily for 7 days) to reduce inflammation and promote healing. This therapeutic approach resulted in ulcer regression without complications; early identification and timely intervention are essential for optimal patient outcomes.

This case report highlights an effective treatment strategy for chemotherapy-induced neutropenic ulcers. It provides practical insights for healthcare professionals managing oral complications in patients with cancer and contributes to the clinical understanding of neutropenic ulcer evaluation and treatment. Despite various available therapeutic strategies, evidence supporting their efficacy remains variable across patient populations. Future clinical research is required to optimize treatment protocols, compare different inventions, and explore novel management strategies for chemotherapy-associated oral ulcerations.

The novelty in this case lies in distinguishing neutropenic ulcers as a specific subset of chemotherapy-related oral mucosal damage. While oral mucositis is well-documented, this report emphasizes the need for early clinical recognition of neutropenic ulcers, especially in patients with severe neutropenia, to guide timely interventions. Furthermore, although corticosteroids are commonly used for oral mucositis, their role in neutropenic ulcers remains poorly established. This case demonstrates successful ulcer regression following treatment with triamcinolone acetonide-based paste in Orabase, suggesting topical corticosteroids as a potential first-line therapy in this subset of patients. In addition, the adjunctive use of chlorhexidine 0.02% mouthwash was beneficial owing to its bactericidal, fungicidal, and virucidal properties. This aligns with emerging research suggesting that modulation of the oral microbiome may influence mucositis severity and ulcer healing. Overall, this case provides a real-world example of an effective, noninvasive treatment strategy that can be integrated in routine oncology care.

## Conclusion

This case report highlights the oral complications associated with chemotherapy, emphasizing the importance of vigilant monitoring and timely intervention in managing neutropenic ulcers. The use of topical or systemic corticosteroids, antiseptics, or antibiotics has proven effective in reducing inflammation and promoting healing. Early identification and prompt treatment of neutropenic ulcers are crucial for preventing complications and ensuring patient outcomes.

## Data Availability

The datasets generated during the current study are available from the corresponding author on reasonable request.

## Abbreviations

AML: Acute myelogenous leukemia
ANC: Absolute neutrophil count
CBC: Complete blood count
NFkB: Nuclear transcription factor kappa B
OM: Oral mucositis
ROS: Reactive oxygen species
TNFα: Tumor necrosis factor-alpha

## References

[1] Howell DA, McCaughan D, Smith AG, Patmore R, Roman E. Incurable but treatable: understanding, uncertainty and impact in chronic blood cancers—a qualitative study from the UK’s Haematological Malignancy Research Network. PLoS ONE. 2022;17(2): e0263672. 35143569 10.1371/journal.pone.0263672 PMC8830712

[2] Papakonstantinou E, Dragoumani K, Efthimiadou A, Palaiogeorgou AM, Pierouli K, Mitsis T, *et al*. Haematological malignancies implications during the times of the COVID-19 pandemic (Review). Oncol Lett. 2021;22(6):856. 34777590 10.3892/ol.2021.13117 PMC8581473

[3] Pérez GB, Calaf GM, Villalba MTM, Prieto KS, Burgos FC. Frequency of hematologic malignancies in the population of Africa, Chile. Oncol Lett. 2019;18(5):5637–43. 31612068 10.3892/ol.2019.10858 PMC6781717

[4] Hungria V, Chiattone C, Pavlovsky M, Abenoza L, Agreda G, Armenta J, *et al*. Epidemiology of hematologic malignancies in real-world settings: findings from the hemato-oncology Latin America observational registry study. J Glob Oncol. 2019;5:1–19. 10.1200/JGO.19.00025 PMC6882510 31774711

[5] Waghmare TP, Prabhat DP, Keshan P, Vaideeswar P. An autopsy study of hematolymphoid malignancies. Int J Res Med Sci. 2019;7(4):9.

[6] Krok-Schoen JL, Fisher JL, Stephens JA, Mims A, Ayyappan S, Woyach JA, *et al*. Incidence and survival of hematological cancers among adults ages ≥75 years. Cancer Med. 2018;7(7):3425–33. 29654631 10.1002/cam4.1461 PMC6051144

[7] Egesie O, Agaba P, Silas O, Achenbach C, Zoakah A, Agbaji O, *et al*. Presentation and survival in patients with hematologic malignancies in Jos, Nigeria: a retrospective cohort analysis. J Med Trop. 2018;20(1):49–56. 29963503 10.4103/jomt.jomt_8_18 PMC6024253

[8] Chennamadhavuni A, Lyengar V, Shimanovsky A. Leukemia. StatPearls; 2022. 32809325

[9] Gomes AOF, Silva Junior A, Noce CW, Ferreira M, Maiolino A, Torres SR. The frequency of oral conditions detected in hematology inpatients. Hematol Transfus Cell Ther. 2018;40(3):240–4. 30128432 10.1016/j.htct.2018.02.006 PMC6098180

[10] Stana P, Marina G, Anca D. Oral manifestations in acute leukemia as the first sign. Interdiscip Approach Diagn Treat. 2015;2:186–92.

[11] Amjad MT, Chidharla A, Kasi A. Cancer Chemotherapy. StatPearls. Treasure Island (FL) ineligible companies. Disclosure: Anusha Chidharla declares no relevant financial relationships with ineligible companies. Disclosure: Anup Kasi declares no relevant financial relationships with ineligible companies; 2023.

[12] Velten DB, Zandonade E, Monteiro de Barros Miotto MH. Prevalence of oral manifestations in children and adolescents with cancer submitted to chemotherapy. BMC Oral Health. 2017;17(1):49. 28109192 10.1186/s12903-016-0331-8 PMC5251332

[13] Poulopoulos A, Papadopoulos P, Andreadis D. Chemotherapy: oral side effects and dental interventions – a review of the literature. Stomatol Dis Sci. 2017;1:35–49.

[14] Visconti R, Della Monica R, Grieco D. Cell cycle checkpoint in cancer: a therapeutically targetable double-edged sword. J Exp Clin Cancer Res. 2016;35(1):153. 27670139 10.1186/s13046-016-0433-9 PMC5037895

[15] Goyri BLM, Ramos MEC, Pérez EE. Estomatotoxicidad bucal inducida por quimioterapia. Revista Odontológica Mexicana. 2014;18(2):89–95.

[16] Thirumaran R, Prendergast GC, Gilman PB. Chapter 7—cytotoxic chemotherapy in clinical treatment of cancer. In: Prendergast GC, Jaffee EM, editors. Cancer immunotherapy. Burlington: Academic Press; 2007. p. 101–16.

[17] Carey PJ. Drug-induced myelosuppression: diagnosis and management. Drug Saf. 2003;26(10):691–706. 12862504 10.2165/00002018-200326100-00003

[18] Jena S, Hasan S, Panigrahi R, Das P, Mishra N, Saeed S. Chemotherapy-associated oral complications in a south Indian population: a cross-sectional study. J Med Life. 2022;15(4):470–8. 35646189 10.25122/jml-2021-0342 PMC9126462

[19] Muhammad R, Alzubaidee A. Oral complications of cancer medication in hemato-oncologic patients. Diyala J Med. 2020;19:180–91.

[20] Finch GL, Burns-Naas LA. Cancer chemotherapeutic agents. In: Wexler P, editor. Encyclopedia of toxicology. 3rd ed. Oxford: Academic Press; 2014. p. 630–41.

[21] Sakong Y, Choi MK, Lee JH. The impact of chemotherapy-induced neutropenia on the outcome of direct-to-implant immediate breast reconstruction. Ann Palliat Med. 2021;10(5):5181–7. 33894733 10.21037/apm-21-508

[22] Okunaka M, Kano D, Matsui R, Kawasaki T, Uesawa Y. Comprehensive analysis of chemotherapeutic agents that induce infectious neutropenia. Pharmaceuticals (Basel). 2021;14(7):681. 34358105 10.3390/ph14070681 PMC8308812

[23] Tang C, Li MH, Chen YL, Sun HY, Liu SL, Zheng WW, *et al*. Chemotherapy-induced niche perturbs hematopoietic reconstitution in B-cell acute lymphoblastic leukemia. J Exp Clin Cancer Res. 2018;37(1):204. 30157922 10.1186/s13046-018-0859-3 PMC6114852

[24] Wang Y, Liu L, Pazhanisamy SK, Li H, Meng A, Zhou D. Total body irradiation causes residual bone marrow injury by induction of persistent oxidative stress in murine hematopoietic stem cells. Free Radical Biol Med. 2010;48(2):348–56. 19925862 10.1016/j.freeradbiomed.2009.11.005 PMC2818724

[25] Crawford J. Improving the management of chemotherapy-induced neutropenia. J Support Oncol. 2004;2(2 Suppl 2):36–9. 16108418

[26] Waladkhani AR. Pegfilgrastim: a recent advance in the prophylaxis of chemotherapy-induced neutropenia. Eur J Cancer Care (Engl). 2004;13(4):371–9. 15305906 10.1111/j.1365-2354.2004.00503.x

[27] Fuchs O. Introductory chapter: development of neutrophils and their role in hematopoietic microenvironment regulation. In: Ota F, Seyyed Shamsadin A, editors. Cells of the immune system. Rijeka: IntechOpen; 2020. p. Ch. 1.

[28] Kruger P, Saffarzadeh M, Weber AN, Rieber N, Radsak M, von Bernuth H, *et al*. Neutrophils: between host defence, immune modulation, and tissue injury. PLoS Pathog. 2015;11(3): e1004651. 25764063 10.1371/journal.ppat.1004651 PMC4357453

[29] Kim MH, Yang D, Kim M, Kim SY, Kim D, Kang SJ. A late-lineage murine neutrophil precursor population exhibits dynamic changes during demand-adapted granulopoiesis. Sci Rep. 2017;7(1):39804. 28059162 10.1038/srep39804 PMC5216372

[30] Herron C. Know your WBCs. Nurs Made Incredibly Easy. 2012;10(1):11–5.

[31] Lehman HK, Segal BH. The role of neutrophils in host defense and disease. J Allergy Clin Immunol. 2020;145(6):1535–44. 32283205 10.1016/j.jaci.2020.02.038 PMC8912989

[32] Zecha J, Raber-Durlacher JE, Laheij A, Westermann AM, Epstein JB, de Lange J, *et al*. The impact of the oral cavity in febrile neutropenia and infectious complications in patients treated with myelosuppressive chemotherapy. Support Care Cancer. 2019;27(10):3667–79. 31222393 10.1007/s00520-019-04925-8 PMC6726710

[33] Morais EF, Lira JA, Macedo RA, Santos KS, Elias CT, Morais ML. Oral manifestations resulting from chemotherapy in children with acute lymphoblastic leukemia. Braz J Otorhinolaryngol. 2014;80(1):78–85. 24626896 10.5935/1808-8694.20140015 PMC9443976

[34] Napenas JJ, Brennan MT, Bahrani-Mougeot FK, Fox PC, Lockhart PB. Relationship between mucositis and changes in oral microflora during cancer chemotherapy. Oral Surg Oral Med Oral Pathol Oral Radiol Endod. 2007;103(1):48–59. 17178494 10.1016/j.tripleo.2005.12.016

[35] Helei NI, Helei VM, Zhulkevych IV. Secondary lesions of the mucous membrane of the oral cavity as a side effect of complex anticancer treatment: a literature review. J Med Life. 2023;16(11):1585–90. 38406781 10.25122/jml-2023-0060 PMC10893569

[36] Subramaniam P, Babu KL, Nagarathna J. Oral manifestations in acute lymphoblastic leukemic children under chemotherapy. J Clin Pediatr Dent. 2008;32(4):319–24. 18767465 10.17796/jcpd.32.4.0p1462t621w20477

[37] Vucicevic Boras V, Vidovic Juras D, Aurer I, Basic-Kinda S, Mikulic M. Gingival ulcerations in a patient with acute myeloid leukemia: a case report and literature review. Acta Clin Croat. 2019;58(3):556–60. 31969772 10.20471/acc.2019.58.03.23 PMC6971802

[38] Pulito C, Cristaudo A, Porta C, Zapperi S, Blandino G, Morrone A, *et al*. Oral mucositis: the hidden side of cancer therapy. J Exp Clin Cancer Res. 2020;39(1):210. 33028357 10.1186/s13046-020-01715-7 PMC7542970

[39] Napenas JJ, Shetty KV, Streckfus CF. Oral mucositis: review of pathogenesis, diagnosis, prevention, and management. Gen Dent. 2007;55(4):335–44 (**quiz 45-6, 76**). 17682645

[40] Compilato D, Cirillo N, Termine N, Kerr AR, Paderni C, Ciavarella D, *et al*. Long-standing oral ulcers: proposal for a new ‘S-C-D classification system.’ J Oral Pathol Med. 2009;38(3):241–53. 19141062 10.1111/j.1600-0714.2008.00722.x

[41] Sonis ST. Mucositis as a biological process: a new hypothesis for the development of chemotherapy-induced stomatotoxicity. Oral Oncol. 1998;34(1):39–43. 9659518 10.1016/s1368-8375(97)00053-5

[42] Sciuca AM, Neamţu M, Marcu D, Costan V, Popa C. Oral mucositis in patients with chemotherapy treatment. Roman J Oral Rehabil. 2024;16:452–60.

[43] Al Beesh FAZ, Martini N, Suleiman S, Aljoujou A. Oral manifestations associated with neutropenia in Syrian patients diagnosed with hematological malignancies and undergoing chemotherapy: a cross-sectional study. Medicine. 2024;103(2): e36780. 38215147 10.1097/MD.0000000000036780 PMC10783351

[44] Kaul R, David CM, Savitha G, Rema J, Ramnarayan K, Sanjay CJ, *et al*. Case report drug induced neutropenia manifesting as oral ulcerations. J Indian Acad Oral Med Radiol. 2022;21(2):72–5.

[45] Cotomacio CC, Magliano GC, Alves FA, Simoes A. Local management of neutropenic ulcer in a patient under breast cancer treatment. Photodiagn Photodyn Ther. 2020;32: 101997. 10.1016/j.pdpdt.2020.101997 32961326

[46] Fontanella C, Bolzonello S, Lederer B, Aprile G. Management of breast cancer patients with chemotherapy-induced neutropenia or febrile neutropenia. Breast Care (Basel). 2014;9(4):239–45. 25404882 10.1159/000366466 PMC4209284

[47] Ono K, Ueno T, Kido MA, Hitomi S, Naniwa M, Nakatomi C, *et al*. Recent advances in the treatment of oral ulcerative mucositis from clinical and basic perspectives. J Oral Biosci. 2024;66(3):504–10. 38866365 10.1016/j.job.2024.06.002

[48] Omara MS, Abdullah WH, Abd El-Bary NM, El Madbouh G, Elkalashy R. Effect of chlorhexidine mouthwash on chemotherapy-induced oral mucositis among patients with cancer. J Menoufia Nurs J. 2024;9(4):125–42.

[49] Arvanitidou IE, Nikitakis NG, Sklavounou A. Oral manifestations of T-cell large granular lymphocytic leukemia: a case report. J Oral Maxillofacial Res. 2011;2(3): e4. 10.5037/jomr.2011.2304 PMC3886074 24421996

[50] Copete MA, Sheridan DP. Large granular lymphocyte leukemia and its association with oral neutropenic ulcerations: a case report. Oral Surg Oral Med Oral Pathol Oral Radiol Endod. 2000;90(4):474–7. 11027385 10.1067/moe.2000.107972

